# Poly[bis(μ_3_-thio­cyanato-κ^3^ *N*:*S*:*S*′)(μ_2_-thio­cyanato-κ^2^ *N*:*S*)(4′-*p*-tolyl-2,2′:6′,2′′-terpyridine-κ^3^ *N*,*N*′,*N*′′)cadmium(II)silver(I)]

**DOI:** 10.1107/S160053681003744X

**Published:** 2010-09-30

**Authors:** Yu-Yang Li, Zhen-Hong Wei, Seik Weng Ng

**Affiliations:** a Department of Chemistry, Nanchang University, Nanchang 330031, People’s Republic of China; b Department of Chemistry, University of Malaya, 50603 Kuala Lumpur, Malaysia

## Abstract

The title compound, [AgCd(NCS)_3_(C_22_H_17_N_3_)]_*n*_, is a hetero­atom ribbon coordination polymer. The central Cd atom is chelated by the 4′-*p*-tolyl-2,2′:6′,2′′-terpyridine ligand and is coordinated by the N atoms of three thio­cyanate ions in an octa­hedral geometry whereas the Ag atom is coordinated by the four S atoms of four thio­cyanate ions in a distorted tetra­hedral geometry. Of the three thio­cyanate ions, one functions in a *μ*_2_-bridging mode and two in a *μ*_3_-bridging mode. The ribbon coordination polymer propagates along the *a*-axis.

## Related literature

For the synthesis and coordination chemistry of the ter­pyridine ligand, see: Zhang *et al.* (2006 ▶).
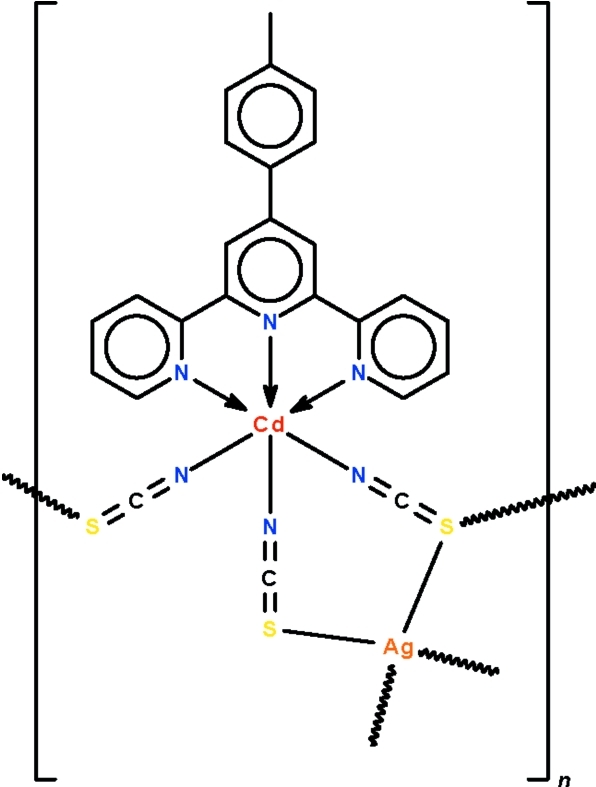


## Experimental

### 

#### Crystal data

[AgCd(NCS)_3_(C_22_H_17_N_3_)]
*M* *_r_* = 717.90
Triclinic, 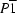

*a* = 10.2431 (10) Å
*b* = 10.7881 (10) Å
*c* = 13.1180 (12) Å
α = 73.045 (2)°
β = 69.000 (2)°
γ = 88.231 (2)°
*V* = 1290.1 (2) Å^3^
*Z* = 2
Mo *K*α radiation
μ = 1.85 mm^−1^
*T* = 295 K
0.30 × 0.30 × 0.25 mm

#### Data collection

Bruker SMART diffractometer
Absorption correction: multi-scan (*SADABS*; Sheldrick, 1996 ▶) *T*_min_ = 0.604, *T*_max_ = 1.000
6870 measured reflections
4432 independent reflections
3910 reflections with *I* > 2σ(*I*)
*R*_int_ = 0.018

#### Refinement

*R*[*F* ^2^ > 2σ(*F* ^2^)] = 0.037
*wR*(*F* ^2^) = 0.097
*S* = 1.02
4432 reflections
326 parameters
H-atom parameters constrained
Δρ_max_ = 0.75 e Å^−3^
Δρ_min_ = −0.59 e Å^−3^

### 

Data collection: *SMART* (Bruker, 2003 ▶); cell refinement: *SAINT* (Bruker, 2003 ▶); data reduction: *SAINT*; program(s) used to solve structure: *SHELXS97* (Sheldrick, 2008 ▶); program(s) used to refine structure: *SHELXL97* (Sheldrick, 2008 ▶); molecular graphics: *X-SEED* (Barbour, 2001 ▶); software used to prepare material for publication: *publCIF* (Westrip, 2010 ▶).

## Supplementary Material

Crystal structure: contains datablocks global, I. DOI: 10.1107/S160053681003744X/nk2062sup1.cif

Structure factors: contains datablocks I. DOI: 10.1107/S160053681003744X/nk2062Isup2.hkl

Additional supplementary materials:  crystallographic information; 3D view; checkCIF report

## Figures and Tables

**Table 1:** Selected bond lengths (Å)

Cd1—N1	2.344 (3)
Cd1—N2	2.326 (3)
Cd1—N3	2.322 (3)
Cd1—N4	2.308 (4)
Cd1—N5	2.312 (4)
Cd1—N6	2.275 (4)
Ag1—S1	2.707 (2)
Ag1—S1^i^	2.589 (1)
Ag1—S2	2.639 (1)
Ag1—S3^ii^	2.521 (1)

Symmetry codes: (i) 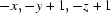; (ii) 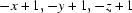.

**Fig. 1.**
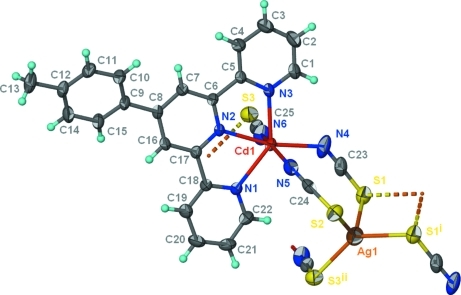
Displacement ellipsoid plot of a portion of the ribbon structure of [AgCd(NCS)3(C22H17N3)]n at the 50% probability level; hydrogen atoms are drawn as spheres of arbitrary radius. Symmetry codes: (i) -x, -y+1, -z+1; (ii) -x+1, -y+1, -z+1.

**Fig. 2.**
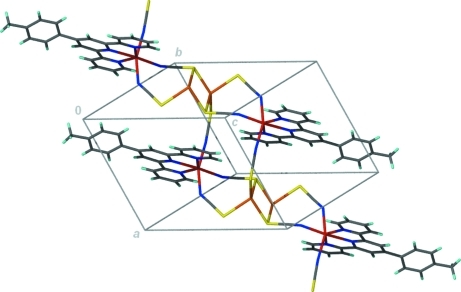
The ribbon coordination polymer in the title compound, which propagates in the a-axis direction.

## supplementary crystallographic information

### Comment

We have recently explored the coordination chemistry of 4'-aryl-2,2':6',2"-terpyridines; such neutral ligands feature three pyridyl sites that are capable of terdentate chelation (Zhang *et al.*, 2006). Occasionally, we have been able to synthesize a bis-chelated metal system whose positive charge is balanced by a metallate ion. In the present study, the attempt at synthesizing bis(4'-*p*-tolyl-2,2':6',2"-terpyridine)cadmium tristhiocyanatoargentate gave instead a compound formulated from the diffraction analaysis as [AgCd(NCS)_3_(C_22_H_17_N_3_)]*_n_* (Scheme I, Fig. 1). The heteroatom coordination polymer has the Cd centre coordinated by the 4'-*p*-tolyl-2,2':6',2"-terpyridine ligand and the N atoms of three thiocyanate ions in an octahedral geometry. The Ag atom is coordinated by the S atoms of four thiocyanate ions in a tetrahedral geometry. Of the three thiocynate ions, one functions in a µ_2_-bridging mode and two in a µ_3_-bridging mode. The ribbon coordination polymer propagates along the *a*-axis of the triclinic unit cell. (Fig. 2).

### Experimental

Silver thiocyanate (0.066 g, 0.4 mmol), cadmium perchlorate hexahydrate (0.042 g, 0.1 mmol) and 4'-*p*-tolyl-2,2':6',2"-terpyridine (0.065 g, 0.2 mmol, which was synthesized by using a literature procedure (Zhang *et al.*, 2006), along with triphenylphosphine (0.105, 0.4 mmol) and acetonitrile (8 ml) were placed in a 15-ml, Teflon-lined, stainless-steel Parr bomb. The reactor was heated in an oven at 723 K for 72 h. It was then cooled to room temperature at a rate of 10 K an hour. Yellow crystals were obtained in 50% (based on 4'-*p*-tolyl-2,2':6',2"-terpyridine).

### Refinement

Carbon-bound H-atoms were placed in calculated positions (C—H 0.93 to 0.96 Å) and were included in the refinement in the riding model approximation, with *U*(H) set to 1.2 to 1.5*U*(C).

### Crystal data

**Table:**

[AgCd(NCS)_3_(C_22_H_17_N_3_)]	*Z* = 2
*M**_r_* = 717.90	*F*(000) = 704
Triclinic, *P*1	*D*_x_ = 1.848 Mg m^−^^3^
Hall symbol: -P 1	Mo *K*α radiation, λ = 0.71073 Å
*a* = 10.2431 (10) Å	Cell parameters from 2780 reflections
*b* = 10.7881 (10) Å	θ = 2.7–25.0°
*c* = 13.1180 (12) Å	µ = 1.85 mm^−^^1^
α = 73.045 (2)°	*T* = 295 K
β = 69.000 (2)°	Prism, yellow
γ = 88.231 (2)°	0.30 × 0.30 × 0.25 mm
*V* = 1290.1 (2) Å^3^	

### Data collection

**Table:**

Bruker SMART diffractometer	4432 independent reflections
Radiation source: fine-focus sealed tube	3910 reflections with *I* > 2σ(*I*)
graphite	*R*_int_ = 0.018
φ and ω scans	θ_max_ = 25.0°, θ_min_ = 2.1°
Absorption correction: multi-scan (*SADABS*; Sheldrick, 1996)	*h* = −9→12
*T*_min_ = 0.604, *T*_max_ = 1.000	*k* = −10→12
6870 measured reflections	*l* = −14→15

### Refinement

**Table:**

Refinement on *F*^2^	Primary atom site location: structure-invariant direct methods
Least-squares matrix: full	Secondary atom site location: difference Fourier map
*R*[*F*^2^ > 2σ(*F*^2^)] = 0.037	Hydrogen site location: inferred from neighbouring sites
*wR*(*F*^2^) = 0.097	H-atom parameters constrained
*S* = 1.02	*w* = 1/[σ^2^(*F*_o_^2^) + (0.0548*P*)^2^ + 0.5516*P*] where *P* = (*F*_o_^2^ + 2*F*_c_^2^)/3
4432 reflections	(Δ/σ)_max_ = 0.001
326 parameters	Δρ_max_ = 0.75 e Å^−^^3^
0 restraints	Δρ_min_ = −0.59 e Å^−^^3^

### Fractional atomic coordinates and isotropic or equivalent isotropic displacement parameters (Å^2^)

**Table:**

	*x*	*y*	*z*	*U*_iso_*/*U*_eq_	
Cd1	0.35097 (3)	0.60113 (3)	0.72815 (2)	0.03873 (12)	
Ag1	0.10392 (4)	0.65042 (5)	0.43358 (4)	0.06953 (15)	
S1	0.15459 (13)	0.40610 (14)	0.53373 (12)	0.0620 (3)	
S2	0.02307 (13)	0.81055 (13)	0.55890 (11)	0.0564 (3)	
S3	0.67482 (14)	0.24010 (13)	0.73061 (11)	0.0581 (3)	
N1	0.5157 (3)	0.7499 (3)	0.5680 (3)	0.0406 (8)	
N2	0.4812 (3)	0.7228 (3)	0.7841 (3)	0.0326 (7)	
N3	0.2798 (3)	0.5376 (3)	0.9263 (3)	0.0399 (8)	
N4	0.1985 (5)	0.4575 (5)	0.7163 (5)	0.0832 (15)	
N5	0.1895 (4)	0.7448 (4)	0.6917 (4)	0.0584 (10)	
N6	0.5008 (4)	0.4416 (4)	0.7139 (4)	0.0665 (12)	
C1	0.1772 (5)	0.4438 (5)	0.9948 (4)	0.0532 (11)	
H1	0.1315	0.4041	0.9613	0.064*	
C2	0.1365 (5)	0.4039 (5)	1.1107 (4)	0.0603 (13)	
H2	0.0646	0.3385	1.1553	0.072*	
C3	0.2025 (5)	0.4612 (5)	1.1601 (4)	0.0620 (13)	
H3	0.1763	0.4360	1.2393	0.074*	
C4	0.3089 (5)	0.5572 (5)	1.0916 (4)	0.0545 (12)	
H4	0.3553	0.5970	1.1246	0.065*	
C5	0.3470 (4)	0.5943 (4)	0.9750 (3)	0.0337 (8)	
C6	0.4614 (4)	0.6971 (4)	0.8953 (3)	0.0337 (8)	
C7	0.5406 (4)	0.7635 (4)	0.9314 (3)	0.0363 (9)	
H7	0.5251	0.7436	1.0089	0.044*	
C8	0.6438 (4)	0.8600 (4)	0.8517 (3)	0.0340 (8)	
C9	0.7284 (4)	0.9362 (4)	0.8865 (3)	0.0369 (9)	
C10	0.6754 (4)	0.9573 (4)	0.9935 (3)	0.0415 (9)	
H10	0.5896	0.9157	1.0469	0.050*	
C11	0.7497 (5)	1.0396 (4)	1.0209 (4)	0.0459 (10)	
H11	0.7120	1.0524	1.0926	0.055*	
C12	0.8775 (4)	1.1029 (4)	0.9452 (4)	0.0447 (10)	
C13	0.9541 (5)	1.1953 (5)	0.9751 (5)	0.0663 (14)	
H13A	0.9749	1.2780	0.9169	0.099*	
H13B	1.0399	1.1609	0.9801	0.099*	
H13C	0.8963	1.2060	1.0475	0.099*	
C14	0.9318 (4)	1.0779 (4)	0.8406 (4)	0.0503 (11)	
H14	1.0195	1.1170	0.7889	0.060*	
C15	0.8601 (4)	0.9973 (4)	0.8109 (4)	0.0440 (10)	
H15	0.8997	0.9832	0.7398	0.053*	
C16	0.6626 (4)	0.8850 (4)	0.7365 (3)	0.0355 (8)	
H16	0.7299	0.9495	0.6807	0.043*	
C17	0.5810 (4)	0.8137 (4)	0.7051 (3)	0.0323 (8)	
C18	0.5954 (4)	0.8332 (4)	0.5847 (3)	0.0353 (8)	
C19	0.6844 (5)	0.9295 (4)	0.4939 (4)	0.0505 (11)	
H19	0.7387	0.9869	0.5063	0.061*	
C20	0.6929 (5)	0.9406 (5)	0.3842 (4)	0.0595 (13)	
H20	0.7532	1.0052	0.3222	0.071*	
C21	0.6119 (5)	0.8558 (5)	0.3677 (4)	0.0587 (13)	
H21	0.6156	0.8615	0.2945	0.070*	
C22	0.5254 (5)	0.7624 (5)	0.4611 (4)	0.0568 (12)	
H22	0.4704	0.7045	0.4499	0.068*	
C23	0.1773 (5)	0.4366 (4)	0.6421 (5)	0.0560 (12)	
C24	0.1223 (4)	0.7706 (4)	0.6354 (4)	0.0446 (10)	
C25	0.5739 (5)	0.3592 (5)	0.7190 (4)	0.0473 (10)	

### Atomic displacement parameters (Å^2^)

**Table:**

	*U*^11^	*U*^22^	*U*^33^	*U*^12^	*U*^13^	*U*^23^
Cd1	0.04172 (18)	0.04043 (19)	0.03918 (19)	−0.00649 (13)	−0.01985 (14)	−0.01209 (13)
Ag1	0.0610 (3)	0.0831 (3)	0.0699 (3)	0.0058 (2)	−0.0246 (2)	−0.0300 (2)
S1	0.0546 (7)	0.0666 (8)	0.0731 (9)	0.0051 (6)	−0.0310 (6)	−0.0237 (7)
S2	0.0565 (7)	0.0618 (8)	0.0601 (7)	0.0134 (6)	−0.0275 (6)	−0.0245 (6)
S3	0.0647 (8)	0.0528 (7)	0.0564 (7)	0.0111 (6)	−0.0235 (6)	−0.0148 (6)
N1	0.0443 (19)	0.046 (2)	0.0326 (18)	−0.0096 (15)	−0.0156 (15)	−0.0100 (15)
N2	0.0344 (16)	0.0323 (17)	0.0337 (17)	−0.0058 (13)	−0.0143 (14)	−0.0105 (13)
N3	0.0363 (17)	0.0424 (19)	0.0394 (18)	−0.0107 (14)	−0.0115 (15)	−0.0115 (15)
N4	0.095 (4)	0.065 (3)	0.126 (4)	−0.002 (3)	−0.077 (4)	−0.035 (3)
N5	0.050 (2)	0.055 (2)	0.065 (3)	−0.0041 (18)	−0.025 (2)	−0.004 (2)
N6	0.059 (3)	0.065 (3)	0.088 (3)	0.015 (2)	−0.032 (2)	−0.035 (3)
C1	0.048 (2)	0.056 (3)	0.053 (3)	−0.018 (2)	−0.016 (2)	−0.014 (2)
C2	0.049 (3)	0.060 (3)	0.054 (3)	−0.017 (2)	−0.003 (2)	−0.008 (2)
C3	0.069 (3)	0.064 (3)	0.039 (3)	−0.018 (3)	−0.007 (2)	−0.009 (2)
C4	0.063 (3)	0.064 (3)	0.033 (2)	−0.021 (2)	−0.012 (2)	−0.013 (2)
C5	0.0331 (19)	0.033 (2)	0.035 (2)	−0.0038 (15)	−0.0117 (16)	−0.0111 (16)
C6	0.037 (2)	0.034 (2)	0.032 (2)	−0.0043 (16)	−0.0150 (16)	−0.0101 (16)
C7	0.038 (2)	0.041 (2)	0.031 (2)	−0.0049 (17)	−0.0113 (17)	−0.0130 (17)
C8	0.0305 (18)	0.034 (2)	0.041 (2)	0.0000 (15)	−0.0157 (17)	−0.0133 (17)
C9	0.038 (2)	0.032 (2)	0.048 (2)	−0.0030 (16)	−0.0234 (18)	−0.0136 (18)
C10	0.046 (2)	0.039 (2)	0.044 (2)	−0.0021 (18)	−0.0191 (19)	−0.0146 (19)
C11	0.059 (3)	0.043 (2)	0.049 (3)	0.002 (2)	−0.030 (2)	−0.020 (2)
C12	0.050 (2)	0.032 (2)	0.067 (3)	0.0007 (18)	−0.037 (2)	−0.015 (2)
C13	0.068 (3)	0.048 (3)	0.107 (4)	0.000 (2)	−0.054 (3)	−0.031 (3)
C14	0.037 (2)	0.048 (3)	0.065 (3)	−0.0087 (19)	−0.021 (2)	−0.011 (2)
C15	0.037 (2)	0.049 (3)	0.050 (3)	−0.0034 (18)	−0.0166 (19)	−0.019 (2)
C16	0.0336 (19)	0.033 (2)	0.038 (2)	−0.0075 (16)	−0.0108 (16)	−0.0091 (17)
C17	0.0331 (19)	0.033 (2)	0.034 (2)	0.0000 (15)	−0.0140 (16)	−0.0124 (16)
C18	0.0357 (19)	0.039 (2)	0.031 (2)	−0.0022 (16)	−0.0124 (16)	−0.0102 (17)
C19	0.060 (3)	0.047 (3)	0.042 (2)	−0.014 (2)	−0.017 (2)	−0.010 (2)
C20	0.076 (3)	0.055 (3)	0.033 (2)	−0.016 (2)	−0.011 (2)	−0.001 (2)
C21	0.076 (3)	0.069 (3)	0.030 (2)	−0.005 (3)	−0.019 (2)	−0.011 (2)
C22	0.070 (3)	0.066 (3)	0.040 (3)	−0.013 (2)	−0.026 (2)	−0.015 (2)
C23	0.051 (3)	0.036 (2)	0.089 (4)	0.000 (2)	−0.039 (3)	−0.014 (2)
C24	0.039 (2)	0.037 (2)	0.045 (2)	−0.0052 (18)	−0.002 (2)	−0.0088 (19)
C25	0.052 (3)	0.052 (3)	0.045 (2)	−0.004 (2)	−0.020 (2)	−0.021 (2)

### Geometric parameters (Å, °)

**Table:**

Cd1—N1	2.344 (3)	C5—C6	1.490 (5)
Cd1—N2	2.326 (3)	C6—C7	1.379 (5)
Cd1—N3	2.322 (3)	C7—C8	1.391 (5)
Cd1—N4	2.308 (4)	C7—H7	0.9300
Cd1—N5	2.312 (4)	C8—C16	1.396 (5)
Cd1—N6	2.275 (4)	C8—C9	1.477 (5)
Ag1—S1	2.707 (2)	C9—C10	1.396 (6)
Ag1—S1^i^	2.589 (1)	C9—C15	1.398 (5)
Ag1—S2	2.639 (1)	C10—C11	1.386 (5)
Ag1—S3^ii^	2.521 (1)	C10—H10	0.9300
S1—C23	1.642 (6)	C11—C12	1.376 (6)
S1—Ag1^i^	2.5885 (13)	C11—H11	0.9300
S2—C24	1.636 (5)	C12—C14	1.387 (6)
S3—C25	1.630 (5)	C12—C13	1.503 (6)
S3—Ag1^ii^	2.5206 (14)	C13—H13A	0.9600
N1—C22	1.336 (5)	C13—H13B	0.9600
N1—C18	1.345 (5)	C13—H13C	0.9600
N2—C17	1.339 (5)	C14—C15	1.375 (6)
N2—C6	1.343 (5)	C14—H14	0.9300
N3—C1	1.339 (5)	C15—H15	0.9300
N3—C5	1.351 (5)	C16—C17	1.390 (5)
N4—C23	1.153 (7)	C16—H16	0.9300
N5—C24	1.153 (5)	C17—C18	1.484 (5)
N6—C25	1.147 (6)	C18—C19	1.374 (6)
C1—C2	1.359 (6)	C19—C20	1.379 (6)
C1—H1	0.9300	C19—H19	0.9300
C2—C3	1.354 (7)	C20—C21	1.366 (6)
C2—H2	0.9300	C20—H20	0.9300
C3—C4	1.376 (6)	C21—C22	1.363 (6)
C3—H3	0.9300	C21—H21	0.9300
C4—C5	1.370 (5)	C22—H22	0.9300
C4—H4	0.9300		
			
N6—Cd1—N4	84.66 (17)	C7—C6—C5	123.5 (3)
N6—Cd1—N5	160.41 (16)	C6—C7—C8	120.0 (3)
N4—Cd1—N5	81.73 (16)	C6—C7—H7	120.0
N6—Cd1—N3	92.25 (14)	C8—C7—H7	120.0
N4—Cd1—N3	97.42 (16)	C7—C8—C16	117.4 (3)
N5—Cd1—N3	103.41 (14)	C7—C8—C9	121.9 (3)
N6—Cd1—N2	95.23 (13)	C16—C8—C9	120.7 (3)
N4—Cd1—N2	167.09 (16)	C10—C9—C15	117.5 (3)
N5—Cd1—N2	101.17 (13)	C10—C9—C8	121.1 (3)
N3—Cd1—N2	69.67 (10)	C15—C9—C8	121.3 (4)
N6—Cd1—N1	91.04 (15)	C11—C10—C9	120.6 (4)
N4—Cd1—N1	123.62 (16)	C11—C10—H10	119.7
N5—Cd1—N1	84.88 (13)	C9—C10—H10	119.7
N3—Cd1—N1	138.95 (11)	C12—C11—C10	122.0 (4)
N2—Cd1—N1	69.28 (11)	C12—C11—H11	119.0
S3^ii^—Ag1—S1^i^	138.17 (5)	C10—C11—H11	119.0
S3^ii^—Ag1—S2	105.98 (4)	C11—C12—C14	117.1 (4)
S1^i^—Ag1—S2	90.37 (4)	C11—C12—C13	121.1 (4)
S3^ii^—Ag1—S1	108.43 (4)	C14—C12—C13	121.7 (4)
S1^i^—Ag1—S1	95.96 (4)	C12—C13—H13A	109.5
S2—Ag1—S1	118.55 (4)	C12—C13—H13B	109.5
C23—S1—Ag1^i^	114.50 (18)	H13A—C13—H13B	109.5
C23—S1—Ag1	96.64 (17)	C12—C13—H13C	109.5
Ag1^i^—S1—Ag1	84.04 (4)	H13A—C13—H13C	109.5
C24—S2—Ag1	99.72 (16)	H13B—C13—H13C	109.5
C25—S3—Ag1^ii^	98.82 (16)	C15—C14—C12	122.1 (4)
C22—N1—C18	118.6 (3)	C15—C14—H14	118.9
C22—N1—Cd1	122.4 (3)	C12—C14—H14	118.9
C18—N1—Cd1	118.8 (2)	C14—C15—C9	120.7 (4)
C17—N2—C6	119.9 (3)	C14—C15—H15	119.7
C17—N2—Cd1	120.1 (2)	C9—C15—H15	119.7
C6—N2—Cd1	120.0 (2)	C17—C16—C8	120.1 (3)
C1—N3—C5	118.3 (3)	C17—C16—H16	120.0
C1—N3—Cd1	122.7 (3)	C8—C16—H16	120.0
C5—N3—Cd1	119.0 (2)	N2—C17—C16	121.0 (3)
C23—N4—Cd1	134.6 (5)	N2—C17—C18	115.5 (3)
C24—N5—Cd1	140.6 (4)	C16—C17—C18	123.5 (3)
C25—N6—Cd1	172.6 (4)	N1—C18—C19	120.8 (3)
N3—C1—C2	123.3 (4)	N1—C18—C17	116.1 (3)
N3—C1—H1	118.4	C19—C18—C17	123.1 (3)
C2—C1—H1	118.4	C18—C19—C20	119.7 (4)
C3—C2—C1	118.7 (4)	C18—C19—H19	120.1
C3—C2—H2	120.6	C20—C19—H19	120.1
C1—C2—H2	120.6	C21—C20—C19	119.3 (4)
C2—C3—C4	119.1 (4)	C21—C20—H20	120.3
C2—C3—H3	120.4	C19—C20—H20	120.3
C4—C3—H3	120.4	C22—C21—C20	118.3 (4)
C5—C4—C3	120.3 (4)	C22—C21—H21	120.9
C5—C4—H4	119.9	C20—C21—H21	120.9
C3—C4—H4	119.9	N1—C22—C21	123.4 (4)
N3—C5—C4	120.3 (3)	N1—C22—H22	118.3
N3—C5—C6	116.4 (3)	C21—C22—H22	118.3
C4—C5—C6	123.3 (3)	N4—C23—S1	177.5 (5)
N2—C6—C7	121.7 (3)	N5—C24—S2	177.7 (4)
N2—C6—C5	114.8 (3)	N6—C25—S3	178.1 (5)
			
S3^ii^—Ag1—S1—C23	100.41 (18)	C1—N3—C5—C4	−0.8 (6)
S1^i^—Ag1—S1—C23	−114.05 (18)	Cd1—N3—C5—C4	−178.8 (3)
S2—Ag1—S1—C23	−20.36 (19)	C1—N3—C5—C6	179.4 (4)
S3^ii^—Ag1—S1—Ag1^i^	−145.53 (4)	Cd1—N3—C5—C6	1.3 (5)
S1^i^—Ag1—S1—Ag1^i^	0.0	C3—C4—C5—N3	0.4 (7)
S2—Ag1—S1—Ag1^i^	93.69 (5)	C3—C4—C5—C6	−179.8 (4)
S3^ii^—Ag1—S2—C24	−82.97 (15)	C17—N2—C6—C7	0.7 (6)
S1^i^—Ag1—S2—C24	136.05 (15)	Cd1—N2—C6—C7	177.2 (3)
S1—Ag1—S2—C24	39.05 (15)	C17—N2—C6—C5	179.8 (3)
N6—Cd1—N1—C22	87.0 (4)	Cd1—N2—C6—C5	−3.7 (4)
N4—Cd1—N1—C22	2.8 (4)	N3—C5—C6—N2	1.5 (5)
N5—Cd1—N1—C22	−73.8 (4)	C4—C5—C6—N2	−178.3 (4)
N3—Cd1—N1—C22	−178.3 (3)	N3—C5—C6—C7	−179.4 (4)
N2—Cd1—N1—C22	−177.7 (4)	C4—C5—C6—C7	0.7 (6)
N6—Cd1—N1—C18	−98.6 (3)	N2—C6—C7—C8	0.3 (6)
N4—Cd1—N1—C18	177.1 (3)	C5—C6—C7—C8	−178.7 (4)
N5—Cd1—N1—C18	100.6 (3)	C6—C7—C8—C16	−0.3 (6)
N3—Cd1—N1—C18	−4.0 (4)	C6—C7—C8—C9	178.2 (4)
N2—Cd1—N1—C18	−3.4 (3)	C7—C8—C9—C10	−27.7 (6)
N6—Cd1—N2—C17	89.3 (3)	C16—C8—C9—C10	150.8 (4)
N4—Cd1—N2—C17	178.2 (6)	C7—C8—C9—C15	156.8 (4)
N5—Cd1—N2—C17	−79.9 (3)	C16—C8—C9—C15	−24.7 (6)
N3—Cd1—N2—C17	179.7 (3)	C15—C9—C10—C11	2.2 (6)
N1—Cd1—N2—C17	0.2 (3)	C8—C9—C10—C11	−173.4 (4)
N6—Cd1—N2—C6	−87.2 (3)	C9—C10—C11—C12	−0.3 (6)
N4—Cd1—N2—C6	1.7 (8)	C10—C11—C12—C14	−1.9 (6)
N5—Cd1—N2—C6	103.6 (3)	C10—C11—C12—C13	177.9 (4)
N3—Cd1—N2—C6	3.2 (3)	C11—C12—C14—C15	2.3 (6)
N1—Cd1—N2—C6	−176.3 (3)	C13—C12—C14—C15	−177.6 (4)
N6—Cd1—N3—C1	−85.5 (4)	C12—C14—C15—C9	−0.3 (7)
N4—Cd1—N3—C1	−0.6 (4)	C10—C9—C15—C14	−1.9 (6)
N5—Cd1—N3—C1	82.6 (4)	C8—C9—C15—C14	173.7 (4)
N2—Cd1—N3—C1	179.7 (4)	C7—C8—C16—C17	−0.6 (6)
N1—Cd1—N3—C1	−179.7 (3)	C9—C8—C16—C17	−179.1 (4)
N6—Cd1—N3—C5	92.4 (3)	C6—N2—C17—C16	−1.7 (6)
N4—Cd1—N3—C5	177.3 (3)	Cd1—N2—C17—C16	−178.2 (3)
N5—Cd1—N3—C5	−99.4 (3)	C6—N2—C17—C18	179.2 (3)
N2—Cd1—N3—C5	−2.3 (3)	Cd1—N2—C17—C18	2.7 (4)
N1—Cd1—N3—C5	−1.7 (4)	C8—C16—C17—N2	1.7 (6)
N6—Cd1—N4—C23	−88.6 (6)	C8—C16—C17—C18	−179.3 (3)
N5—Cd1—N4—C23	77.3 (6)	C22—N1—C18—C19	0.3 (6)
N3—Cd1—N4—C23	179.9 (6)	Cd1—N1—C18—C19	−174.3 (3)
N2—Cd1—N4—C23	−178.7 (5)	C22—N1—C18—C17	−179.4 (4)
N1—Cd1—N4—C23	−0.9 (7)	Cd1—N1—C18—C17	6.0 (5)
N6—Cd1—N5—C24	5.1 (8)	N2—C17—C18—N1	−5.7 (5)
N4—Cd1—N5—C24	−41.3 (5)	C16—C17—C18—N1	175.3 (4)
N3—Cd1—N5—C24	−137.1 (5)	N2—C17—C18—C19	174.6 (4)
N2—Cd1—N5—C24	151.4 (5)	C16—C17—C18—C19	−4.5 (6)
N1—Cd1—N5—C24	83.7 (5)	N1—C18—C19—C20	−0.3 (7)
C5—N3—C1—C2	0.6 (7)	C17—C18—C19—C20	179.4 (4)
Cd1—N3—C1—C2	178.6 (4)	C18—C19—C20—C21	0.3 (8)
N3—C1—C2—C3	−0.1 (8)	C19—C20—C21—C22	−0.2 (8)
C1—C2—C3—C4	−0.3 (8)	C18—N1—C22—C21	−0.2 (7)
C2—C3—C4—C5	0.2 (8)	Cd1—N1—C22—C21	174.1 (4)

Symmetry codes: (i) −*x*, −*y*+1, −*z*+1; (ii) −*x*+1, −*y*+1, −*z*+1.
